# Metabolomics Studies in Psoriatic Disease: A Review

**DOI:** 10.3390/metabo11060375

**Published:** 2021-06-10

**Authors:** John Koussiouris, Nikita Looby, Melanie Anderson, Vathany Kulasingam, Vinod Chandran

**Affiliations:** 1 Schroeder Arthritis Institute, Krembil Research Institute, University Health Network, Toronto, ON M5T 0S8, Canada; john.koussiouris@uhnresearch.ca (J.K.); nikita.looby@uhnresearch.ca (N.L.); 2 Department of Laboratory Medicine and Pathobiology, University of Toronto, Toronto, ON M5T 0S8, Canada; vathany.kulasingam@uhn.ca; 3 Library and Information Services, University Health Network, Toronto, ON M5T 0S8, Canada; melanie.anderson@uhn.ca; 4 Division of Clinical Biochemistry, Laboratory Medicine Program, University Health Network, Toronto, ON M5T 0S8, Canada; 5 Division of Rheumatology, Department of Medicine, University of Toronto, Toronto, ON M5S 1A8, Canada; 6 Institute of Medical Science, University of Toronto, Toronto, ON M5S 1A8, Canada; 7 Department of Medicine, Memorial University, St. John’s, NL A1B 3V6, Canada

**Keywords:** metabolomics, metabolites, psoriatic disease, psoriasis, psoriatic arthritis

## Abstract

Metabolomics investigates a broad range of small molecules, allowing researchers to understand disease-related changes downstream of the genome and proteome in response to external environmental stimuli. It is an emerging technology that holds promise in identifying biomarkers and informing the practice of precision medicine. In this review, we summarize the studies that have examined endogenous metabolites in patients with psoriasis and/or psoriatic arthritis using nuclear magnetic resonance (NMR) or mass spectrometry (MS) and were published through 26 January 2021. A standardized protocol was used for extracting data from full-text articles identified by searching OVID Medline ALL, OVID Embase, OVID Cochrane Central Register of Controlled Trials and BIOSIS Citation Index in Web of Science. Thirty-two studies were identified, investigating various sample matrices and employing a wide variety of methods for each step of the metabolomics workflow. The vast majority of studies identified metabolites, mostly amino acids and lipids that may be associated with psoriasis diagnosis and activity. Further exploration is needed to identify and validate metabolomic biomarkers that can accurately and reliably predict which psoriasis patients will develop psoriatic arthritis, differentiate psoriatic arthritis patients from patients with other inflammatory arthritides and measure psoriatic arthritis activity.

## 1. Introduction

Psoriasis (Ps) is an inflammatory skin disease that affects over 7.25 million Americans and 1.25 million Canadians [1]. Chronic plaque Ps, the most common form of Ps, clinically manifests as well-demarcated erythematous scaly plaques affecting any part of the skin. Severe Ps is associated with an increased risk of mortality and several comorbidities [2]. Approximately 25% of Ps patients, have an inflammatory arthritis termed psoriatic arthritis (PsA) [3], which is associated with reduced quality of life and functional capacity [4]. Clinical features of PsA include peripheral joint involvement, inflammatory spinal disease, enthesitis, tendonitis, dactylitis and other extra-articular features [2]. Several comorbidities are common in patients with PsA including obesity, hyperlipidemia and cardiovascular events, all of which are more prevalent in PsA patients than in patients with Ps alone [5]. The pathogenesis of both Ps and PsA is thought to be complex and multifactorial with genetic, immunologic and environmental causes [2]. Psoriatic disease is a term used to encompass the systemic inflammatory involvement of different organs in patients with Ps and/or PsA. Recently, there has been a developing interest in discovering predictive and prognostic biological markers (biomarkers) for psoriatic disease. Biomarker discovery may shed light on unexplored pathobiological mechanisms that drive disease onset and activity and may lead to identifying novel disease targets. Biomarkers are a step towards precision medicine and may allow clinicians to identify subsets of patients that require more strict monitoring or use of more targeted therapies.

Metabolomics is an emerging “omics” science that studies a broad range of small molecules (<1500 Da) present in a biological system. This includes diverse chemical and physical structures such as sugars, nucleotides, amino acids and lipids. The metabolome is a rapid indicator of biological status [6]. Metabolites are constantly changing compared to the genome and transcriptome where changes take more time to take effect. Thus, the metabolome depicts a snapshot of the physiological status rather than a prediction of that status as provided on the genome level. Furthermore, the metabolome is a reflection of the intersection of the genome with the environment and the microbiome [7]. Using metabolomics for biomarker discovery can be advantageous because it accounts for interactions between these factors, some of which if not all, may play a role in disease pathogenesis. Metabolomics allows researchers to understand disease-related changes downstream of the genome and proteome in response to external environmental stimuli. Thus, an organism’s metabolome can be considered the most reflective of its observable phenotype. Metabolomics in combination with other “omics” fields and the interactions between them can be a powerful strategy in identifying biomarkers.

The purpose of this review is to summarize the most recent metabolomics studies in psoriatic disease and determine the gaps in the literature to provide direction for further research exploration. To achieve this goal, we will first introduce the typical metabolomics workflow, summarize and discuss the methods used in each step of the workflow, and finally discuss the results of the studies to identify any gaps in the literature that can provide a direction for future research exploration.

## 2. Metabolomics Workflow

**Figure 1:**
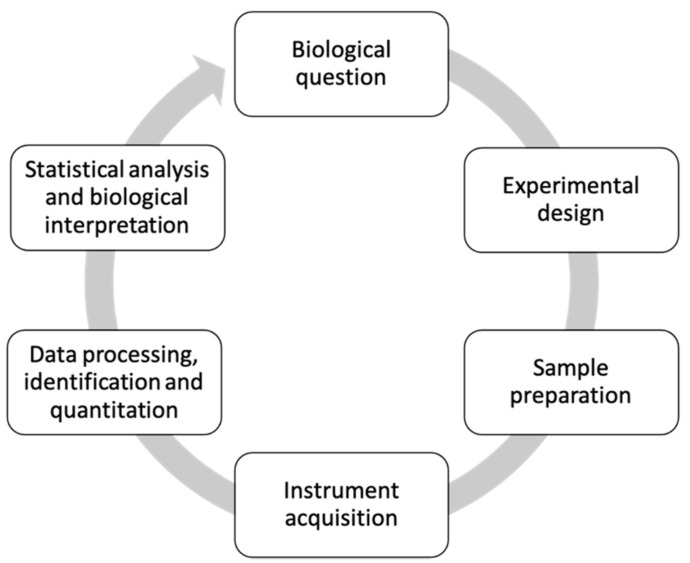
Metabolomics workflow.

Metabolomics studies often follow a standard workflow consisting generally of 5–6 steps (Figure 1). Initially, we will briefly discuss the purpose of each step of the workflow, then in keeping with the workflow outline, we will further review in detail the Ps- and PsA-focused studies in the following sections.

The metabolomics workflow typically begins with the biological question—the purpose and aims of a particular study, which can be hypothesis- or non-hypothesis-driven. The second step consists of the experimental design which addresses how the study will attempt to answer the biological question. Within this step, researchers determine if the study will use a targeted or untargeted approach wherein the latter seeks to analyze all the metabolites of a biological system, as opposed to the former, which focuses on identifying and quantifying select metabolites or classes of metabolites. Sample size, sample type (biological fluids or tissues), and sample collection are also determined within this step. Following experimental design, *sample preparation* techniques are then considered and are dependent on the sample size, type, costs, throughput and resources available. This step is the most critical aspect in the workflow since the type of sample preparation method employed, can have an enormous effect on the observed metabolite profile and in turn, the accuracy of biological interpretation [8]. *Instrumental acquisition* is the fourth step in the workflow wherein the most commonly used analytical techniques for identifying and quantifying metabolites are nuclear magnetic resonance (NMR) or mass spectrometry (MS) which can be paired with either gas chromatography (GC-MS) or liquid chromatography (LC-MS). The final steps—steps 5 and 6—consists of *data processing* and *statistical analysis*. Once metabolomics data has been acquired via the abovementioned instrumentation, these last steps of the metabolomics workflow help to reduce the complexity of the original data files into an understandable and useable data matrix containing enhanced significant metabolite signals across the various sample groups analyzed.

### 2.1. The Biological Question

Generally, psoriatic disease-related metabolomics studies aim to identify metabolic markers that can be used for Ps and/or PsA diagnosis. While a few studies have examined the metabolite profiles of patients with autoimmune diseases (ADs) [9,10], or immune-mediated inflammatory diseases (IMIDs) with no emphasis on Ps or PsA specifically [11], the majority of studies included in this review sought to identify metabolites that were associated with Ps by investigating Ps patients in comparison to healthy volunteers [12,13,14,15,16,17,18,19,20,21,22,23,24,25,26,27,28,29,30,31]. Other studies focused on identifying metabolite profiles in Ps patients and correlating them with different measurements of disease activity [13,19,22,28,32,33]. Another important question in psoriatic disease that has garnered much interest pertains to determining which Ps patients have or will develop PsA. Several studies compared the metabolite profiles of PsA patients with Ps patients and healthy controls to discover metabolomic biomarkers associated with PsA [12,18,31]. Other studies investigated the metabolic differences between patients with PsA and other forms of arthritis such as rheumatoid arthritis (RA) [34,35,36]. Furthermore, it is worth highlighting that due to the heterogenous clinical representations of PsA disease, it is difficult to assess disease activity in PsA patients [37]. Acute phase reactants such as C-reactive protein are within normal range in many patients, rendering them ineffective in accurately measuring PsA activity [37]. Current methods of assessing PsA disease activity are based on semi-objective measures such as joint counts and physician and patient global assessments, which often poorly correlate with one another [37,38]. Additionally, these composite measures place a time and resource burden on patients and clinicians and ultimately may not accurately reflect inflammatory activity. As such, identifying biomarkers that accurately and reliably measure PsA disease activity is becoming increasingly important in order to improve clinical management of the disease. There were a few metabolomics studies that explored the association between the metabolome and PsA activity [39,40,41].

### 2.2. Experimental Design

Most studies included in our literature search were non-hypothesis driven and therefore employed an untargeted metabolomics approach [12,14,15,16,19,20,21,22,23,25,26,31,32,33,36,41,42]. However, there were several studies that analyzed specific metabolites using a targeted metabolomics approach [9,10,13,18,24,27,28,30,39,40]. Fewer studies exploited both approaches to analyze different classes of metabolites [17,29,35], while a couple studies initially performed an untargeted discovery study followed by validation of the results in a targeted way [11,34]. Almost half of the studies we found were preliminary studies with a small sample size of less than fifty subjects, whereas other studies ranged from 90 to as many as 1210 subjects. Many of the studies utilized rigorous inclusion and exclusion criteria for patient selection in order to reduce the impact of potential confounding variables. Patients and controls were often selected so that they had similar distributions of epidemiological variables such as gender, age and BMI. Sample collection variables such as the fasting time of the individual before sample collection and the time of day of sample collection were also routinely standardized. Every study had its own unique exclusion criteria, but most did not include subjects who were pregnant, had any history of other chronic or acute disorders, or were receiving any recent treatment for their disease.

A variety of biological samples including peripheral blood (plasma or serum), urine and tissue were used in these metabolomics studies. The collection, handling and storage of samples are critical steps that can affect the study’s experimental reproducibility [43]. Blood can be processed in one of two ways to produce either plasma or serum. Given the differences between these two biochemical processes, plasma and serum-based studies may provide different metabolite profiles [44]. Serum is derived from blood that has coagulated and is the most commonly archived biological fluid with the majority of metabolomics studies included in this review examining the serum metabolome [9,12,13,14,15,16,17,34,35,39,40]. Contrastingly, plasma does not undergo the coagulation process and thus takes less time to process and contains additional clotting proteins not present in serum [45]. There were fewer studies that investigated metabolites in plasma [18,19,20,21,32,36,42]. Many of the studies that examined metabolites in the blood of patients with psoriatic disease required subjects to fast prior to sample collection. Additionally, there was a single study that looked specifically at mononuclear cells in peripheral blood [31].

Urine, which is readily available, can be easily and inexpensively collected from patients in large volumes in a non-invasive way [44]. The urine metabolome, however, can be dilute and can fluctuate over time according to renal integrity [45]. There were only four studies that analyzed metabolites in the urine [10,11,28,41].

Compared to liquid biological samples, tissue samples are more complex (solid and semi-solid) and thus require the tissue structure to be disrupted and homogenized using manual, physical or mechanical methods prior to metabolite extraction [44]. Obtaining tissue samples is an invasive process but offers researchers insight into tissue directly implicated in the disease being studied and tissue specific metabolites involved. Thus, there were several studies that investigated the skin metabolome of Ps patients [22,23,24,25,26,27].

Comparing metabolites across multiple sample matrices may provide additional information about their role in the disease given their distribution in various sample types. As such, a few studies analyzed both the skin and blood metabolome [29,30,33].

### 2.3. Sample Preparation

Given the ever-changing nature of the metabolome, in order to recover an accurate snapshot of the metabolome at a particular time, metabolic quenching is required to inactivate enzymes that may continue to change the sample once it is removed from its native environment. The objective of sample preparation in metabolomics is to provide an appropriate quenching mechanism to effectively isolate exclusively, all the small molecules of interest from their various complex samples while reducing matrix interferences [8,44]. Untargeted metabolomics require sample preparation procedures that are more unselective in order to capture a broad range of metabolites, whereas targeted metabolomics studies employ methods that extract specific classes of metabolites [8]. Sample preparation is especially important for MS-based metabolomics platforms that are paired with separation technologies like GC and LC as these techniques require significant sample clean-up to avoid clogging, fouling or damaging of these instruments. Although various sample preparation methods exist for chromatographic-paired MS-based studies, the metabolomics studies included in this review often employed one of the two following types of sample preparation strategies for peripheral blood and urine, each with their own advantages and disadvantages: liquid-liquid extraction (LLE) and solid-phase extraction (SPE).

LLE separates compounds based on their solubilities in two different immiscible liquids [46], and is a popular technique in metabolomics due to its simplicity, speed, fast method optimization and lack of specialized equipment [44]. Traditional LLE approaches such as the Bligh−Dyer and Folch are the most popular extraction methods particularly for lipids [47]. While several studies used LLE for sample preparation [9,10,18,24,28,31,34,36,42], the method is burdened by its large solvent-to-sample ratio, use of environmentally toxic organic solvents and is not amenable to automation [47]. The selectivity and clean-up offered by LLE is also low compared to other methods [44]. Solid-liquid extraction (SLE) is another liquid-partitioning method very similar to LLE that isolates analytes from solid and semi-solid matrices. A couple of studies utilized this method to extract metabolites from skin samples [25,27]. Protein precipitation (PPt) is another widely used sample preparation method for biological fluids that can sometimes be categorized as an LLE method since it requires usually the addition of cold solvent to allow the precipitation of proteins from the sample, which are then effectively removed, leaving only the metabolites [44]. PPt shares many of the same benefits and limitations as LLE [44], and the majority of studies included in this review used PPt to isolate metabolites and prepare the sample before analysis [13,15,16,17,19,20,21,29,30,32,33,36,39,40,42].

Solid-phase extraction (SPE) is another sample preparation method used in metabolomics that uses a solid adsorbent contained in a cartridge to extract analytes from a sample [8]. Compared to LLE, SPE uses less environmentally toxic solvents, provides excellent sample clean-up and can be more easily automated [8,44]. The main disadvantage with SPE is that the choice of solid sorbent increases selectivity for certain metabolites, resulting in reduced coverage of a broader range of metabolite classes [8]. Thus, SPE is more commonly used in targeted metabolomics studies rather than untargeted studies. Another limitation of SPE is that it involves specialized equipment and extensive preparatory procedures [44]. SPE was the next most common sample preparation method following LLE and PPt [23,29,30,31,36,40].

Unlike biological fluids, tissue sample harvesting and processing, are more involved and usually necessitate additional steps such as homogenization and/or lyophilization prior to use of any of the above-mentioned strategies [43]. A few studies exploring the skin metabolome used a specialized biocompatible hydrogel micropatch probe to sample skin [22,33].

In contrast to chromatographic-paired MS-based platforms, NMR-based metabolomics platforms do not require extensive sample preparation methods. The NMR-based metabolomics studies included in this review either had a simple centrifugation step or diluted the sample with a phosphate buffer [11,14,35,41], while a couple of studies did not include any sample preparation methods at all [12,26].

### 2.4. Instrument Acquisition

NMR and mass spectrometry (MS) paired with gas chromatography (GC-MS) or liquid chromatography (LC-MS) are the most commonly used analytical tools to perform metabolomics. It is important to note that that no single analytical technique can detect all metabolites in a sample due to the extensive range of metabolite classes, structures, and physicochemical properties. Metabolomics requires a wide array of instrumentation and some studies may employ a combination of methods to broaden and improve metabolite coverage; each method having benefits and limitations (see Table 1) [7].

NMR is a beneficial analytical technique used in metabolomics due to its good quantitative capability in determining absolute concentrations of metabolites with high reproducibility, its minimal sample preparation requirements and ultimately sample conservation since samples are not destroyed [7,50]. NMR works to determine the molecular structures of metabolites in samples by observing the interaction of nuclear spins in the presence of a powerful magnetic field [48,50].

Data is acquired relatively quickly and can be interpreted using established libraries for metabolite comparison [48]. A few limitations of NMR include very high start-up costs, low sensitivity, large sample volume requirements and limited metabolite coverage since it can only detect organic compounds in liquid and solid samples [48]. Despite its limitations, NMR has been used in five metabolomics studies in psoriatic disease [11,14,26,35,41].

Mass spectrometry on the other hand measures the molecular weight of an analyte in relation to its charge (mass-to-charge [*m*/*z*] ratio). MS is commonly paired with either GC or LC separation techniques in metabolomics due to its high sensitivity and good coverage of metabolites [50]. GC-MS allows for the detection of volatile and semi-volatile metabolites from gases and liquids [7], whereas LC-MS is able to detect most semi- and non-volatile organic and some inorganic molecules in liquid and solid samples [48]. Additional benefits of MS platforms include smaller sample volume requirements and lower start-up costs in comparison to NMR [48]. MS, however, like all other analytical techniques is not without its limitations. Chromatographic-paired MS-based systems require extensive sample preparation, destroy the sample, have a slower analysis time and have lower reproducibility compared to NMR [48,50]. Furthermore, absolute quantitation of metabolites is only possible using calibration curves typically with internal standard correction and only a fraction of all metabolites have MS spectra readily available for comparison and identification [48]. Tandem mass spectrometry (MS/MS) acquires additional information about specific metabolites by selecting them for an additional fragmentation step. The majority of studies included in this review used LC-MS based platforms [13,16,17,19,20,21,23,24,25,27,28,30,32,33,39,40], while much fewer number of studies employed GC-MS to analyze metabolites [9,10,12,15]. Other studies employed both LC-MS and GC-MS as their analytical techniques [18,29,34]. Kishikawa et al. and Kishikawa et al. used conventional LC-MS as well as paired mass spectrometry with capillary electrophoresis (CE-MS) [36,42]. Kapoor et al. used ion exchange chromatography to separate charged amino acids [41], and two studies used a customized nanospray desorption electrospray ionization (nanoDESI) MS system [22,33].

### 2.5. Data Processing and Statistical Analysis

After the samples are analyzed by the respective instrumentation, the data files need to be converted and processed prior to statistical analysis [51]. Initial steps of pre-processing NMR data involve apodization, Fourier transform, phasing, baseline correction, chemical shift calibration and normalization [51]. Correction for peak shifts observed due to changes in pH, temperature, or fluctuations in the magnetic field is also performed as well as removal of interfering spectral regions related to possible contaminants in the samples [51]. The pre-processing steps for MS data include peak detection, peak deconvolution, alignment, gap filling and normalization, followed by QC analysis and correction for instrumental drifts [51]. Pre-processing NMR and MS data generates a final table of features with study samples and their corresponding spectral data points and m/z–retention time (RT) pairs, respectively. Once clean and normalized metabolomics data has been generated, pre-treatment can center, scale and transform the data to prepare it for statistical analysis [51,52]. Numerous software was used by studies in this review to pre-process and pre-treat data, including commercial platforms such as MarkerLynx, Progenesis QI or MasterHands, as well as open-source platforms, such as XCMS, MZmine and R.

For NMR-based data, metabolites are identified based on their spectra and chemical shift (ppm) and are quantified based on the area of the peak associated with each metabolite since this represents its concentration [53]. For MS-based data, however, unknown metabolites are identified by their retention time, accurate mass and fragment ion patterns via MS/MS [43]. Unlike NMR, untargeted MS-based metabolomics compares the relative levels of all metabolites in a sample and is unable to absolutely quantify these metabolites. This can be remedied via the use of internal standard correction and calibration curves for absolute quantitation and is typically employed for targeted MS-based metabolomics that uses MS/MS. In regard to statistical analysis, a variety of univariate and multivariate approaches are available to analyze the data. Our review of the literature found several software platforms being used for statistical analysis, with the most common being MetaboAnalyst, SPSS, SIMCA-P and R. The wide variety of tools required to process and model data and the assortment of software platforms available to perform these steps, make it challenging to harmonize this aspect of the metabolomics workflow, potentially contributing to difficulties in reproducibility.

## 3. Results

### 3.1. Studies in Peripheral Blood

#### 3.1.1. Serum

The serum metabolome of Ps and PsA patients and its association with disease diagnosis and disease activity has been investigated in several recent studies (Table 2). Multiple studies revealed drastic differences in amino acid levels in Ps patients compared to healthy controls [12,13,14,15,17]. Bilgic et al. found Ps skin disease activity correlated with metabolic derivatives of the amino acid arginine [13]. Other studies found abnormal levels of carboxylic acids, acylcarnitines, phosphatidylcholines and other organic compounds in the serum of Ps patients compared to healthy volunteers [15,17]. Moreover, Li et al. discovered 44 potential biomarkers for Ps diagnosis, involved in glycerophospholipid metabolism, sphingolipid metabolism, arachidonic acid metabolism and bile acid biosynthesis [16]. Tsoukalas et al. measured abnormal levels of serum fatty acids in patients with autoimmune diseases (ADs) [9]. Although this study looked at the metabolic profile of patients with a variety of ADs, these potential biomarkers are promising and should be further investigated specifically in patients with psoriatic disease. Taken holistically, these studies indicate that various lipid and amino acid metabolic pathways are dysregulated in patients with Ps.

Metabolomics studies in psoriatic disease have also explored how serum metabolic biomarkers may be able to differentiate the diagnosis of Ps, PsA and RA. Armstrong et al. found PsA patients had different levels of glucuronic acid compared to controls and PsA patients also had lower levels of alphaketoglutaric acid and increased lignoceric acid compared to Ps patients [12]. By comparing the metabolites in PsA and RA patients, two research groups found differences in the concentrations of amino acids, organic compounds and lipid ratios [34,35]. Metabolomics studies in serum have also examined how serum metabolite levels correlate with disease activity in PsA patients. Pro-inflammatory and anti-inflammatory eicosanoids correlated with joint disease score in PsA patients [40]. Furthermore, trimethylamine-N-oxide (TMAO) was found to significantly correlate with measures of disease activity for both skin and peripheral joints [39]. An elevated level of TMAO has been indicated as a risk factor for atherosclerosis, cardiovascular disease and obesity [39], the prevalence of which are significantly higher in PsA patients than in patients with Ps alone [5].

#### 3.1.2. Plasma

Several studies have investigated plasma metabolites in Ps patients and their association with Ps diagnosis and activity (Table 3). Several studies described amino acids at different levels in Ps patients compared to healthy controls [19,20,32,42]. Correlation network analyses revealed that psoriasis-associated metabolites centered around the amino acid aspartate [42]. After treatment with the anti-TNFα drug etanercept, the metabolic profiles of severe Ps patients shifted towards that of healthy controls [32]. Additionally, a study by Chen et al. revealed that levels of various carnitines differentiated Ps patients from healthy controls [19]. Several classes of metabolites have been associated with the degree of Ps activity. Two research groups found amino acids and carnitine metabolites correlated with the severity of Ps [19,32]. Plasma nucleotides have also been investigated as metabolite biomarkers for autoimmune and autoinflammatory diseases such as RA, SLE and PsA. Several nucleotides differentiated RA patients and healthy controls, however there were no significant differences between PsA patients and healthy controls [36].

Multiple studies have also reported plasma lipid metabolites to be possible biomarkers for Ps diagnosis. Several phospholipids were identified at drastically different concentrations in Ps patients compared to heathy controls [18,20,21]. The lysoglycerophospholipids LPC and LPA found in Ps patients are inflammatory lipids thought to be involved in several immune-mediated diseases and autoimmune diseases [21]. Li et al. also found elevated levels of fatty acids and other lipid species in Ps patients [20]. Ambrozewicz et al. found reduced fatty acids and elevated lipid peroxidation products and endocannabinoids in patients with Ps and PsA [18]. These fatty acids were also significantly reduced in PsA patients compared to Ps patients. Similarly, a recent study by Kishikawa et al. attributed metabolite differences between PsA and Ps to be primarily from saturated fatty acids [42]. The findings of these studies indicate disturbances in phospholipid and polyunsaturated fatty acid (PUFA) metabolism in the plasma of patients with psoriatic disease.

#### 3.1.3. Mononuclear Cells

Wójcik et al. investigated oxidative lipid modifications in mononuclear cells, mainly lymphocytes isolated from patients suffering from Ps or PsA (Table 4) [31]. Higher 8-isoPGF2a and 4-HNE were seen in PsA patients, whereas 4-HNE-His adducts were higher in Ps patients [31]. The authors also found increased levels of eicosanoids in Ps and PsA patients [31].

### 3.2. Studies in Skin

The skin metabolome of Ps and PsA patients and its association with disease diagnosis and disease activity has been investigated in several recent studies (Table 5). Several studies compared the metabolomic profiles of lesional (PS-L) and non-lesional (PS-NL) skin extracted from Ps patients as well as samples from healthy controls. Pohla et al. found elevated amino acids, acylcarnitines, phospholipids and biogenic amines in PS-L skin compared to healthy controls [25]. Higher levels of amino acids were found in PS-L skin when compared to PS-NL skin [25]. Sugars and amino acids were found at different levels in PS-L and PS-NL skin and these differences depended on whether or not patients improved after topical corticosteroid treatment [26]. The metabolite changes found in patients with good therapeutic effect resembled the metabolite levels in uninvolved skin. The association between metabolites and disease activity in Ps patients has also been investigated in skin. Dutkiewicz et al. found that amino acids and their derivatives correlated with plaque severity scores and differentiated PS-L, PS-NL and healthy skin [22].

Various lipids have been described at different levels in the skin of Ps patients compared to healthy controls. Mathers et al. described increased concentrations of electrophilic fatty acids in lesional skin compared to non-lesional skin, extracted from Ps patients, suggesting that electrophilic fatty acids may be involved in skin inflammation [24]. Luczaj et al. found that the fibroblasts and keratinocytes in Ps patients contained different levels of various ceramides compared to healthy controls [23]. Ceramides are important lipid metabolites that play a crucial role in the formation and maintenance of the skin barrier [23]. Furthermore, Takeichi et al. found hepoxilins and their related lipids were more abundant in psoriatic skin than in normal controls [27]. Lipids produced in the lipoxygenase pathway of arachidonic acid were elevated in Ps skin whereas the lipids produced in the cyclooxygenase pathway were decreased in Ps skin, suggesting that an imbalance between these two pathways may contribute to the pathobiological mechanisms of Ps [27].

### 3.3. Studies in Urine

A few studies seeking to identify biomarkers for disease diagnosis and disease activity in patients with psoriatic disease, have explored metabolites in urine samples (Table 6). Two studies identified biomarkers for disease diagnosis and disease activity in patients with immune-mediated inflammatory diseases (IMIDs) and autoimmune diseases (ADs). Alonso et al. found several metabolites differentiating Ps and PsA patients from healthy controls, with PsA patients experiencing higher disease activity, having lower levels of citrate [11]. A similar study by Tsoukalas et al. revealed significant differences in the levels of several organic acids in patients with IMIDs and ADs [10]. Although these studies looked at the metabolic profile of patients with a variety of diseases, these potential biomarkers are promising and should be further investigated specifically in patients with Ps and PsA.

Setkowicz et al. compared the urinary metabolites of 12(S)-hydroxyeicosatetraenoic (HETE) acid in Ps patients and found higher concentrations of tetranor-12(S)-HETE in Ps patients compared to controls [28]. 12(S)-HETE is an unusual product of 12-lipoxygenation of arachidonic acid produced locally by psoriatic keratinocytes and neutrophils infiltrating psoriatic lesions [28]. In Ps patients, tetranor-12(S)-HETE may be a marker for activation of granulocytes participating in skin inflammation. Kapoor et al. identified changes in the urinary levels of organic acids and organic compounds in patients with RA and PsA before and after treatment with the anti-tumor necrosis factor α (anti-TNFα) agents infliximab and etanercept [41]. The different metabolic profiles after treatment with infliximab and etanercept suggest that these two biological agents may act through distinct mechanisms of action. Future studies examining the metabolic profile of PsA patients who respond and do not respond to these agents may help predict treatment response.

### 3.4. Studies in Multiple Matrices

A few metabolomics studies in psoriatic disease have examined metabolite profiles in multiple sample matrices (Table 7). Comparing metabolite concentrations in different biological millieu may provide additional information about their use as potential biomarkers. Several metabolites in the eicosapentaenoic (EPA), docosahexaenoic (DHA) and arachidonic acid (AA) pathways were elevated in the metabolome of Ps skin and peripheral blood [29,30]. Choline levels in both skin and plasma correlated positively with the severity of Ps, while citrulline in both sample matrices correlated negatively [33]. Furthermore, choline and citrulline in skin and blood showed dynamic changes corresponding to resolution of Ps due to treatment with biologics [33]. The changes of these skin metabolites were more prominent in the responders to the treatment than in the non-responders.

## 4. Discussion

### 4.1. Psoriasis Diagnosis

The majority of metabolomics studies in psoriatic disease herein reviewed focused on discovering metabolites associated with Ps. Differing levels of several amino acids between Ps patients and healthy controls was described. There were some inconsistencies in the literature regarding the levels of asparagine and tryptophan in peripheral blood of Ps patients compared to healthy controls. One study described Ps patients having higher levels of asparagine in serum [15], whereas two studies described reduced levels in serum [12], and plasma [19]. Elevated levels of tryptophan in the plasma of Ps patients were found in one study [20], whereas another found a reduction of the metabolite in serum [14]. These conflicting results may be due to the different analytical techniques used or differing concentrations of metabolites in plasma versus serum. Comparing metabolite levels in the skin in addition to the blood, may provide additional information about a metabolite’s role as a potential biomarker. The amino acids leucine and phenylalanine have been revealed to be elevated in both the peripheral blood and skin lesions extracted from Ps patients, compared to healthy volunteers [15,20,25]. Glutamine has been found at lower levels in the serum and plasma of Ps patients in three separate studies [12,14,19]. On the other hand, lesional skin extracted from Ps patients had higher levels of glutamine compared to non-lesional skin and skin from healthy controls [25]. Both methionine and arginine were found to be reduced in the serum of Ps patients but elevated in psoriatic lesions compared to non-lesional skin extracts [14,25].

Numerous studies have suggested the essential nutrient choline and its derivatives may be potential biomarkers for psoriasis and two studies found a higher relative abundance of choline in skin lesions of Ps patients compared to uninvolved skin [22,26]. Phosphatidylcholines (PC) and lysophosphatidylcholines (LPC/LysoPC) are essential phospholipid components of biological membranes that incorporate choline as a headgroup [17,20]. Lower concentrations of several PC metabolites were found in the serum and plasma of Ps patients compared to healthy subjects [17,21], of which higher rates of skin cell proliferation in Ps patients may be the cause. LPCs are considered inflammatory lipids involved in several immune-mediated diseases [21] and have been shown to induce the chemotaxis of immune cells fueling inflammation in the epidermis [17]. Additionally, two studies found elevated LPCs in the plasma of Ps patients [20,21], the increase of which suggests that they may be clinically relevant to psoriasis pathobiology.

Arachidonic acid (AA) is an omega-6 polyunsaturated fatty acid (PUFA), that gives rise to eicosanoid production and abnormalities in AA metabolism were associated with Ps in several studies. AA was found at lower levels in the plasma metabolome of Ps patients compared to controls [20], and three studies found an increase in one or both of the AA metabolites—12- and 15-hydroxyeicosatetraenoic acid (HETE)–in lesional skin compared to skin from healthy individuals [27,29,30]. Additionally, excretion of 12(S)-HETE in the urine was reduced in Ps patients [28]. Leukotrienes, another family of eicosanoids produced in leukocytes by the oxidation of AA, act as inflammatory mediators, regulating immune responses by autocrine and paracrine signaling [16]. Leukotrienes D4 and E3 were elevated in the serum of Ps patients compared to healthy controls [16]. A reduction in plasma AA and increase in derivatives of AA suggest that dysregulated AA metabolism in the skin may be a key feature of psoriasis, thus metabolites in this pathway may act as possible prognostic biomarkers.

Other metabolites that have been found across studies to be potential biomarkers for psoriasis diagnosis are urea and lactic acid. Two studies found higher levels of both metabolites in the serum of Ps patients [15,17], but lactic acid was also found at significantly lower levels in involved skin of Ps patients than noninvolved skin [22].

### 4.2. Psoriasis Activity

Our literature search revealed inconsistent results regarding the correlation of glutamine with Ps activity. One study described a negative correlation between serum glutamine and PASI scores [32], while another study suggested plasma glutamine was positively correlated with PASI [19]. Citrulline is another amino acid that emerged with conflicting results during our evaluation of the literature. Levels of citrulline in the skin were negatively associated with Ps activity in two studies [22,33], but were found to both positively and negatively correlate with Ps activity, when analyzed in plasma [32,33]. Several studies have found choline to positively correlate with Ps activity in both skin lesions and plasma [22,33]. Other metabolites in peripheral blood and skin that were found to be associated with Ps activity include ADMA, l-arginine/ADMA ratio, threonine, ornithine, glutamic acid, asparagine, urocanic acid and palmitoylcarnitine (C16) [13,19,22,32].

### 4.3. Psoriatic Arthritis Diagnosis

The diagnosis of PsA may be improved with metabolic biomarkers that are able to differentiate PsA from healthy controls, patients with Ps and patients with another type of arthritis such as RA. Compared to healthy subjects, PsA patients have elevated levels of glucuronic acid in their serum [12]. PsA patients also have lower levels of alphaketoglutaric acid and increased lignoceric acid compared to patients with Ps [12]. The plasma metabolome of PsA patients has revealed that linoleic acid (18:2) and (18:3) as well as free arachidonic acid (20:4) and docosahexaenoic acid (22:6) are significantly reduced compared to Ps patients [18]. When compared to RA patients, aspartic acid, glutamic acid, histidine, serine, arachidonic acid, cholesterol, threonic acid and 1-monooleoylglycerol were elevated in PsA patients [34]. Glutamine, heptanoic acid, succinate, pseudouridine, inosine, guanosine, arabitol, cystine, cysteine and phosphoric acid were lower in PsA compared to RA [34]. Additionally, the concentrations of various amino acids (alanine, threonine, leucine and valine), organic compounds (acetate, creatine, lactate and choline) and lipid ratios (L3/L1, L5/L1 and L6/L1) differentiated PsA and seronegative RA (negRA) patients [35]. Additionally, the amino acid phenylalanine was reduced in PsA compared to negRA. The studies that have investigated the association between metabolites and PsA diagnosis have described various amino acids and lipids as potential biomarkers for the disease.

### 4.4. Psoriatic Arthritis Activity

Only a few studies have correlated metabolites with measures of PsA disease activity. In serum, trimethylamine-N-oxide (TMAO) a known risk factor for cardiovascular disease and obesity, positively correlated with PsA skin and peripheral joint activity [39]. Additionally, pro-inflammatory eicosanoids PGE2, HXB3 and 6,15-dk,dh,PGF1a and anti-inflammatory eicosanoids 11-HEPE, 12-HEPE and 15-HEPE correlated with joint disease score in PsA patients [40]. Lastly, reduced urinary citrate was found in PsA patients with higher disease activity [11].

### 4.5. Gaps in the Literature and Future Directions

The purpose of this review was to summarize the most recent metabolomics studies in psoriatic disease and use the determined gaps in the literature to propose a direction for future research. The vast majority of studies presented in this review, identified metabolites that may be associated with psoriasis diagnosis and activity. These studies revealed mostly the involvement of amino acids and pathways associated with lipid metabolism to be potentially useful as biomarkers. Many of these findings were similar across different studies using various sample matrices and metabolomics methods. Some findings, however, were not consistent across different studies, which is likely a result of different samples being used and at least partially attributed to the wide variety of methods available for each step of the metabolomics workflow. Experts in the field are currently building a general consensus concerning the minimum reporting standards for metabolomics experiments in order to facilitate experimental replication and enable comparison of data by others [54]. It is also important to note that identifying metabolites is only the first step in the biomarker discovery process. As opposed to NMR-based platforms which intrinsically quantify metabolites, untargeted mass spectrometry-based studies only quantify metabolites relative to one another and thus must be followed up with targeted studies to measure their exact concentration in the sample.

There were very few studies that examined the metabolome of PsA patients and correlated metabolites with PsA diagnosis and PsA activity. Further exploration is needed to identify and validate biomarkers that can accurately and reliably predict which Ps patients will develop PsA, differentiate PsA patients from patients with other inflammatory arthritides and measure PsA activity. The synovial fluid, which is in direct contact with articular cartilage, bone and synoviocytes may provide the best reflection of the biochemical state of the joint under pathophysiological conditions. Although invasive to obtain, synovial fluid may be a promising source of biomarkers for PsA. Additionally, the availability of a wide variety of treatments for psoriatic disease, poses a need for biomarkers that can predict a patient’s response to a particular therapeutic agent. Metabolomic biomarker profiles may lead to personalized treatment plans that can maximize efficacy while minimizing toxicity. Furthermore, the relationship between the metabolome of patients with psoriatic disease and the microbiome and diet can be more closely examined. In a heterogenous disease like PsA, an accurate biomarker-based test will need to include demographic factors such as sex, gender and ethnicity. Integrating metabolomics with other high-throughput-omics technologies such as genomics, transcriptomics, and proteomics will highlight aberrant signaling pathways likely driving psoriatic disease. In combination with emerging technologies, metabolomics has the potential to advance our understanding of psoriatic disease and lead to the development of clinically useful biomarkers specific for this patient population.

## 5. Conclusions

Metabolomics is an emerging technology that holds promise in identifying biomarkers and informing the practice of precision medicine. In this review, we have summarized studies that have examined endogenous metabolites in patients with Ps and/or PsA using NMR or MS. The majority of studies identified amino acids and lipids that may be associated with Ps diagnosis and activity. Further investigation is needed to identify and validate metabolomic biomarkers that can accurately and reliably predict which Ps patients will develop PsA, differentiate PsA patients from patients with other forms of arthritis and measure PsA activity.

## 6. Materials and Methods

**Figure 2:**
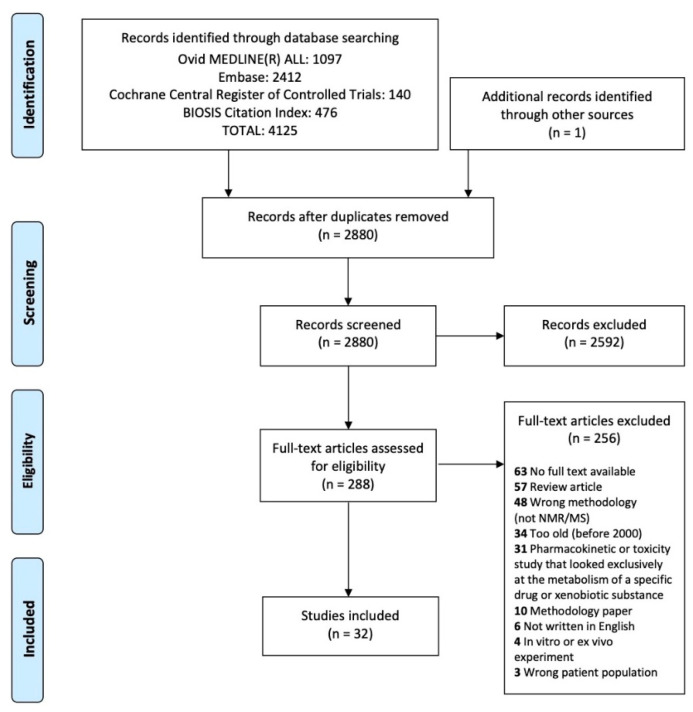
Article screening process.

Our protocol was registered on Open Science Framework on 3 December 2020 [55]. Strategies were developed for four databases, including OVID Medline ALL, OVID Embase, OVID Cochrane Central Register of Controlled Trials and BIOSIS Citation Index in Web of Science, by a health sciences librarian [MA]. The initial results were downloaded 26 January 2021. Appropriate subject headings and keywords for psoriasis and psoriatic arthritis as well as metabolomics and lipidomics were used for each database. Non-human studies were removed; however, no date, age, language or other limits were applied. For complete strategies (see Supplementary File S1). The search strategy yielded 2880 abstracts after duplicates were removed, which were then screened by two members [JK, NL] of the research team based on predetermined inclusion criteria: (1) Study involved subjects with psoriasis and/or psoriatic arthritis; (2) study examined endogenous metabolites in human subjects; (3) study used nuclear magnetic resonance (NMR) or mass spectrometry (MS) as the analytical technique. We excluded case reports, in vitro or ex vivo experiments, methodology papers, review articles, articles published before 2000, articles not written in English and pharmacokinetic or toxicity studies that looked exclusively at the metabolism of specific drug or xenobiotic substance. Two reviewers [JK, NL] independently assessed all titles and abstracts. 288 abstracts were selected for full-text review, full-text articles could not be found for 63 abstracts and 32 articles met all criteria (Figure 2). Data were extracted from the included studies using a piloted standardized form.

## Figures and Tables

**Table 1:** Comparison of commonly employed analytical platforms used in metabolomics.

Features	NMR	GC-MS	LC-MS
Startup Cost	Very high (>$1 million USD)	Modest (~$150,000 USD)	High (>$300,000 USD)
Sample Preparation	Minimal	Required	Required
Sample recovery	Sample recoverable	Sample destroyed	Sample destroyed
Separation	Not required	Required	Usually required
Volume	Large (0.1–0.5 mL)	Modest (0.1–0.2 mL)	Small (10–100 μL)
Analysis time	Fast (2–3 min per sample)	Slow (20–40 min per sample)	Slow (15–40 min per sample)
Sensitivity	Low (LOD = 5 μM)	Good (LOD = 0.5 μM)	Superb (LOD = 0.5 nM)
Selectivity	Mostly nonselective analysis	Selective and nonselective analysis	Selective and nonselective analysis
Quantitation	Inherently quantitative	Quantitative with calibration	Quantitative with calibration
Type	Liquids and solids	Volatile gases and liquids	Liquids and solids
Reproducibility	Higher	Lower	Lower
Analyte range	Detects most organic classes. Cannot detect salts, inorganic ions or non-protonated compounds	Detects most organic and some inorganic molecules	Detects most organic and some inorganic molecules
Data interpretation	Established libraries for comparison	Newer technology, many unknowns	Newer technology, many unknowns

Summarized data from review studies [7,48,49,50]. Abbreviations: GC, gas chromatography; LC, liquid chromatography; LOD, limit of detection; MS, mass spectrometry; NMR, nuclear magnetic resonance.

**Table 2:** Studies in serum.

Study ID	Study Outcome	Study Design	Subjects	Sample Preparation Method	Analytical Techniques	Data Processing Software	Statistical Analysis Software	Results
Armstrong 2014 [12]	Ps diagnosis PsA diagnosis	Untargeted	10 Ps 10 PsA 10 HC	N/A	GC-MS	BinBase	R packages	Ps had higher alpha ketoglutaric acid, lower Asparagine and lower glutamine compared to HC. PsA had higher glucuronic acid compared to HC. PsA had lower alphaketoglutaric acid and increased lignoceric acid compared to Ps.
Bilgic 2015 [13]	Ps diagnosis Ps activity	Targeted	42 Ps 48 HC	PPt	LC-MS	N/A	SPSS	ADMA and homocysteine higher and citrulline and L-arginine/ADMA lower in Ps. PASI scores correlated with ADMA level, L-arginine/ADMA ratio.
Castaldo 2020 [14]	Ps diagnosis	Untargeted	30 Ps 30 HC	Diluted with phosphate buffer	NMR	Chenomx NMR-Suite	MetaboAnalyst	Lower L-tryptophan, L-tyrosine, L-lysine, L-histidine, L-methionine, L-arginine, L-ornithine, and L-glutamine in Ps compared to HC.
Coras 2019a [39]	PsA activity	Targeted	38 PsA	PPt	LC-MS/MS	R	R	TMAO significantly correlated with skin and peripheral joint activity.
Coras 2019b [40]	PsA activity	Targeted	41 PsA	PPt SPE	LC-MS/MS	R	R	Pro-inflammatory eicosanoids PGE2, HXB3 and 6,15-dk, dh, PGF1a and anti-inflammatory eicosanoids 11-HEPE, 12-HEPE and 15-HEPE correlated with joint disease activity. 8,9-diHETrE, 11,12-diHETrE,14,15-diHETrE, 19,20-diHDPA and 7,17 DHDPA negatively correlated with joint disease activity. DHA-anti-inflammatory eicosanoids resolvin D1 and 17-HDoHE, were lower in patients with high disease activity.
Kang 2017 [15]	Ps diagnosis	Untargeted	14 Ps 15 HC	PPt	GC-MS	AMDIS MassHunter	Simca-P	Ps had higher asparagine, aspartic acid, isoleucine, phenylalanine, ornithine, proline, lactic acid and urea. Ps had lower crotonic acid, azelaic acid, ethanolamine and cholesterol.
Li 2017 [16]	Ps diagnosis	Untargeted	75 Ps 75 HC	PPt	LC-MS	MarkerView PeakView	SIMCA-P Hem1 Cytoscape Metaboanlyst	Potential biomarkers were mainly involved in glycerophospholipid metabolism, sphingolipid metabolism, arachidonic acid metabolism and bile acid biosynthesis.
Madsen 2011 [34]	RA diagnosis PsA diagnosis	Untargeted Targeted	39 RA 25 PsA 30 HC	LLE	GC-MS LC-MS	MATLAB ChromaTOF	SIMCA-P+	Aspartic acid, glutamic acid, glutamate, histidine, serine, arachidonic acid, cholesterol, threonic acid, 1-monooleoylglycerol higher in PsA compared to RA. Glutamine, heptanoic acid, succinate, pseudouridine, inosine, guanosine, arabitol, cystine, cysteine and phosphoric acid lower in PsA compared to RA.
Ottas 2017 [17]	Ps diagnosis	Untargeted Targeted	75 Ps 71 HC	PPt	LC-MS	MSConvert XCMS mzMatch.R	R	Acylcarnitines, glutamate, ornithine, phenylalanine, methioninesulfoxide, urea, taurine, phytol and 1,11-undecanedicarboxylic acid higher in Ps. Several phosphatidylcholines lower in Ps.
Souto-Carneiro 2020 [35]	RA diagnosis PsA diagnosis	Untargeted Targeted	49 RA 73 PsA	Diluted with phosphate buffer	NMR	TopSpin	MetaboAnalyst	Increased concentrations of amino acids: alanine, threonine, leucine and valine; organic compounds: acetate, creatine, lactate and choline; and lipid ratios L3/L1, L5/L1 and L6/L1 in PsA compared to negRA. Phenylalanine reduced in PsA compared to negRA.
Tsoukalas 2019 [9]	AD diagnosis	Targeted	240 ADs 163 HC	LLE	GC-MS	N/A	SPSS R	AD had increased levels of C15:1, C20:1n9, C22:1n9, C18:3n3, C18:3n6, and total omega-6/total omega-3 ratio while they had lower levels of total omega-3 fatty acids, C12:0, C17:0, C18:0.

Abbreviations: Ps, psoriasis; PsA, psoriatic arthritis; RA, rheumatoid arthritis; negRA, seronegative rheumatoid arthritis; AD, autoimmune disease; HC, healthy controls; SPE, solid-phase extraction; LLE, liquid-liquid extraction; PPt, protein precipitation; LC-MS, liquid chromatography-mass spectrometry; LC-MS/MS, liquid chromatography-tandem mass spectrometry; GC-MS, gas chromatography-mass spectrometry; NMR, nuclear magnetic resonance.

**Table 3:** Studies in plasma.

Study ID	Study Outcome	Study Design	Subjects	Sample Preparation Method	Analytical Techniques	Data Processing Software	Statistical Analysis Software	Results
Ambrozewicz 2018 [18]	Ps diagnosis PsA diagnosis	Targeted	8 Ps 8 PsA 8 HC	LLE	LC-MS GC-MS	N/A	Stata/IC MetaboAnalyst	Ps and PsA had decreased levels of phospholipids and free polyunsaturated fatty acids. Increased lipid peroxidation products 4-hydroxynonenal, isoprostanes, and neuroprostanes as well as increased levels of endocannabinoids AEA and 2-AG in Ps and PsA.
Chen 2021 [19]	Ps diagnosis Ps activity	Untargeted	45 Ps 45 HC	PPt	LC-MS	Progenesis QI	metaX	Essential amino acids, and branched-chain amino acids increased in Ps. Glutamine, cysteine, and asparagine decreased in Ps. Palmitoylcarnitine (C16) decreased in Ps whereas hexanoylcarnitine (C6) and 3-OH-octadecenoylcarnitine (C18:1-OH) increased in Ps. Glutamine, asparagine, and C16 levels negatively correlated with PASI score in Ps.
Kamleh 2015 [32]	Ps activity	Untargeted	32 mild Ps 32 severe Ps 32 HC	PPt	LC-MS	MSconvert XCMS	R SIMCA-P	Ps-associated perturbations found in three metabolic pathways: (1) arginine and proline, (2) glycine, serine and threonine, and (3) alanine, aspartate, and glutamate. Etanercept treatment shifted the metabolic phenotypes of severe Ps toward that of HC. Circulating metabolite levels pre- and post-Etanercept treatment correlated with PASI score.
Kishikawa 2020 [36]	RA diagnosis SLE diagnosis PsA diagnosis	Untargeted	92 RA 13 SLE 43 PsA 181 HC	PPt LLE SPE	CE-MS LC-MS	MasterHands	R	UTP, ethanolamine phosphate, ATP, GDP, ADP, 6-aminohexanoic acid and taurine increased in RA and xanthine decreased in RA compared to HC. No significant differences in these metabolites between PsA and HC.
Kishikawa 2021 [42]	Ps diagnosis PsA diagnosis	Untargeted	43 PsA 50 Ps 38 HC	PPt LLE	CE-MS LC-MS	MasterHands	R	Ethanolamine phosphate increased in Ps whereas nicotinic acid, and 20α-hydroxyprogesterone decreased, compared to HC. Aspartate was centered on the correlation network among the Ps-associated metabolites. Tyramine significantly increased in PsA than in PsC, whereas mucic acid decreased. Enrichment of vitamin digestion and absorption pathway in Ps compared to PsA. Subnetwork among metabolites formed from saturated fatty acids.
Li 2019 [20]	Ps diagnosis	Untargeted	12 Ps 12 HC	PPt	LC-MS	MarkerLynx	SIMCA-P	Threonine, leucine, phenylalanine, tryptophan, palmitamide, linoleic amide, oleamide, stearamide, cis-11-eicosenamide, trans-13-docosenamide, uric acid, LysoPC (16:0), LysoPC (18:3), LysoPC (18:2), Lys-oPC (18:1) and LysoPC (18:0) higher in Ps. Oleic acid, arachidonic acid and N-linoleoyl taurine lower in Ps.
Zeng 2017 [21]	Ps diagnosis	Untargeted	45 Ps 45 HC	PPt	LC-MS/MS	Progenesis QI metaX	metaX	Higher lysophosphatidicacid, lysophosphatidylcholine and phosphatidic acid in Ps. Lower phosphatidylinositol and phosphatidylcholine in Ps.

Abbreviations: Ps, psoriasis; PsA, psoriatic arthritis; RA, rheumatoid arthritis; SLE, systemic lupus erythematous; HC, healthy controls; SPE, solid-phase extraction; LLE, liquid-liquid extraction; PPt, protein precipitation; LC-MS, liquid chromatography-mass spectrometry; LC-MS/MS, liquid chromatography-tandem mass spectrometry; GC-MS, gas chromatography-mass spectrometry; CE-MS, capillary electrophoresis-mass spectrometry.

**Table 4:** Studies in peripheral blood mononuclear cells.

Study ID	Study Outcome	Study Design	Subjects	Sample Preparation Method	Analytical Techniques	Data Processing Software	Statistical Analysis Software	Results
Wójcik 2019 [31]	Ps diagnosis PsA diagnosis	Untargeted	32 Ps 16 PsA 16 HC	LLE SPE	GC-MS LC-MS/MS	N/A	Statistica	Higher 8-isoPGF2a and 4-HNE in PsA, whereas 4-HNE-His adducts were higher in Ps. Increased eicosanoids in Ps and PsA: PGE1, LTB4, 13HODE, TXB2. Eicosanoids 15-d-PGJ2 and 15-HETE were elevated in Ps and reduced in PsA.

Abbreviations: Ps, psoriasis; PsA, psoriatic arthritis; HC, healthy controls; SPE, solid-phase extraction; LLE, liquid-liquid extraction; LC-MS, liquid chromatography-mass spectrometry; LC-MS/MS, liquid chromatography-tandem mass spectrometry; GC-MS, gas chromatography-mass spectrometry.

**Table 5:** Studies in skin.

Study ID	Study Outcome	Study Design	Subjects	Sample Preparation Method	Analytical Techniques	Data Processing Software	Statistical Analysis Software	Results
Dutkiewicz 2016 [22]	Ps diagnosis Ps activity	Untargeted	100 Ps 100 HC	Hydrogel micropatch probes	nanoDESI MS	Custom C# software	MetaboAnalyst OriginPro	Choline and glutamic acid positively correlated with plaque severity scores whereas urocanic acid and citrulline negatively correlated. The amount of these metabolites in Ps skin were significantly different from HC skin.
Luczaj 2020 [23]	Ps diagnosis	Untargeted	6 Ps 6 HC	SPE	LC-MS/MS	MZmine	MetaboAnalyst	Keratinocytes of Ps patients had higher levels of CER[NS], CER[NP], CER[AS], CER[ADS], CER[AP] and CER[EOS], whereas CER[NDS] was lower in Ps compared to HC. In fibroblasts, Ps patients had higher CER[AS], CER[ADS] and CER[EOS].
Mathers 2018 [24]	Ps diagnosis	Targeted	6 lesional Ps skin 6 non-lesional Ps skin	LLE	LC-MS/MS	N/A	N/A	56% increase in nitro-conjugated linoleic acid (CLA-NO2) levels in lesional skin compared to non-lesional skin, extracted from Ps patients.
Pohla 2020 [25]	Ps diagnosis	Untargeted	20 Ps 19 HC	SLE	LC-MS	N/A	R	Amino acids, acylcarnitines, biogenic amines, lysophosphatidylcholines, phosphatidylcholines, histamine and ADMA increased in Ps lesional samples compared to HC. No significant differences between the metabolite profiles of Ps non-lesional samples and HC skin.
Sitter 2013 [26]	Ps diagnosis	Untargeted	10 Ps	N/A	NMR	PeakFit	SPSS	Lower myo-inositol and glucose, and higher choline and taurine in Ps lesion compared to uninvolved skin. Higher glucose, myo-inositol, GPC and glycine as well as lower choline in patients who improved after corticosteroid treatment versus those that did not improve.
Takeichi 2019 [27]	Ps diagnosis	Targeted	8 Ps/PsA 10 HC	Cryogenic pulverization SLE	LC-MS	N/A	SAS	Most hepoxilins and their related lipids were more abundant in Ps skin than in HC. Lipids produced in the lipoxygenase pathway were elevated in Ps, whereas lipids produced in the cyclooxygenase pathway were reduced in Ps.

Abbreviations: Ps, psoriasis; PsA, psoriatic arthritis; HC, healthy controls; SPE, solid-phase extraction; LLE, liquid-liquid extraction; SLE, solid-liquid extraction; LC-MS, liquid chromatography-mass spectrometry; LC-MS/MS, liquid chromatography-tandem mass spectrometry; NMR, nuclear magnetic resonance.

**Table 6:** Studies in urine.

Study ID	Study Outcome	Study Design	Subjects	Sample Preparation Method	Analytical Techniques	Data Processing Software	Statistical Analysis Software	Results
Alonso 2016 [11]	IMID diagnosis IMID activity	Untargeted discovery Targeted validation	1210 IMID 100 HC	Centrifugation	NMR	FOCUS	R	Identified and validated 26 biomarkers for IMID diagnosis and 3 biomarkers for IMID activity. Ps and PsA had low lower urine citrate, alanine, methylsuccinate and trigonelline compared to HC. Higher PsA activity associated with lower citrate.
Kapoor 2013 [41]	RA activity PsA activity	Untargeted	16 RA 20 PsA	Centrifugation	NMR Ion exchange chromatography	Prometab (in MatLab)	MatLab R	After 12 weeks of infliximab treatment, RA and PsA had increased hippuric acid, citrate, and lactic acid. Choline, phenylacetic acid, urea, creatine, and methylamine increased after etanercept treatment.
Setkowicz 2015 [28]	Ps diagnosis Ps activity	Targeted	200 Ps 200 HC	LLE	LC-MS/MS	N/A	Statistica	Higher Tetranor-12(S)-HETE and lower 12(S)-HETE in Ps compared to HC. Neither metabolites correlated with the type of disease or severity score.
Tsoukalas 2020 [10]	AD diagnosis	Targeted	241 AD 151 HC	LLE	GC-MS	N/A	SPSS R	Increased 2-hydroxyglutarate and 2-hydroxyisobutyrate in AD. Decreased succinate, methylcitrate, malate, pyroglutamate, 2-hydroxybutyrate, methylmalonate, 4-hydroxyphenylpyruvate in AD. Methylmalonate, 2-Hydroxyglutarate and 2-hydroxybutyrate were proposed as potential biomarkers for AD.

Abbreviations: Ps, psoriasis; PsA, psoriatic arthritis; HC, healthy controls; RA, rheumatoid arthritis; IMID, immune-mediated inflammatory diseases; AD, autoimmune disease; LLE, liquid-liquid extraction; LC-MS, liquid chromatography-mass spectrometry; LC-MS/MS, liquid chromatography-tandem mass spectrometry; GC-MS, gas chromatography-mass spectrometry; NMR, nuclear magnetic resonance.

**Table 7:** Studies in multiple matrices.

Study ID	Study Outcome	Study Design	Subjects	Sample Matrix	Sample Preparation Method	Analytical Techniques	Data Processing Software	Statistical Analysis Software	Results
Dutkiewicz 2020 [33]	Ps activity	Untargeted	17 Ps	Skin Plasma	Skin: Hydrogel micropatches Plasma: PPt	Skin: nano-DESI-MS Plasma: LC-MS	Compass DataAnalysis Custom C#	OriginPro	Choline levels in lesional skin correlated positively with severity of the lesions, while citrulline correlated negatively. Plasma choline levels also correlated positively with the severity of the disease (PASI), while citrulline also correlated negatively. Choline and citrulline in skin and blood showed dynamic changes corresponding to resolution of Ps due to the treatment with biologics.
Sorokin 2018a [29]	Ps diagnosis	Untargeted Targeted	Plasma: 60 Ps 30 HC Skin & serum: 8 Ps 7 HC	Plasma Skin Serum	PPt SPE	LC-MS/MS GC-MS	N/A	Stata/IC	Higher arachidonic acid metabolites, as 8-, 12- and 15-hydroxyeicosatetraenoic acid, in lesional skin compared to nonlesional and skin from HC. 13-hydroxyoctadecadienoic acid increased in lesional skin compared with HC skin. Decreased antioxidant markers of glutathione and g-glutamyl and primary and secondary bile acids in the plasma of Ps patients compared to HC.
Sorokin 2018b [30]	Ps diagnosis	Targeted	7 Ps 7 HC	Plasma Serum Skin	PPt SPE	LC-MS/MS	N/A	Prism Stata/IC SIMCA-P	Increased levels of several metabolites in the EPA, DHA and AA metabolome of Ps skin. RvD5, PDx and aspirin-triggered (AT) forms of lipoxin (LX) were present only in lesional Ps skin whereas protectin D1 was present in non-lesional Ps skin. Expression of EPA, DHA and AA pathway markers in the detected peripheral blood metabolome had a similar trend as for Ps skin with slight difference for HC.

Abbreviations: Ps, psoriasis; HC, healthy controls; PPt, protein precipitation; SPE, solid-phase extraction; LC-MS, liquid chromatography-mass spectrometry; LC-MS/MS, liquid chromatography-tandem mass spectrometry; GC-MS, gas chromatography-mass spectrometry.

## Supplementary Materials

The following are available online at https://www.mdpi.com/article/10.3390/metabo11060375/s1, File S1: Search documentation for review.
