# Supplementary material for: Metabolomics Studies in Psoriatic Disease: A Review
Source: Metabolites. 2021 Jun 10;11(6):375. doi: 10.3390/metabo11060375 (PMC8230373; doi:10.3390/metabo11060375)
Supplement: Supplementary file 1 [file metabolites-11-00375-s001.zip › Supplementary S1 - Search documentation.pdf]

S1: Search documentation for review

Searches run January 26, 2021

- OVID Medline ALL
- OVID Embase Classic + Embase
- OVID Cochrane Central Register of Controlled Trials
- Web of Science BIOSIS Citation Index

Strategies from January 26, 2021

Database(s): **Ovid MEDLINE(R) ALL** 1946 to January 25, 2021
Search Strategy:

#	Searches	Results
1	exp Psoriasis/ [includes Arthritis, Psoriatic/]	40936
2	psorias*.mp.	50905
3	psoriat*.mp.	19603
4	(pustul* adj4 palm*).mp.	1083
5	(pustul* adj4 plantar*).mp.	303
6	(arthrit* adj4 mutilans).mp.	163
7	(pustul* adj4 soles).mp.	28
8	1 or 2 or 3 or 4 or 5 or 6 or 7	57463
9	exp Metabolomics/	17971
10	metabolom*.mp.	41919
11	metabolit*.mp.	286493
12	(metabol* adj4 produc*).mp.	46883
13	(metabol* adj4 waste*).mp.	1176
14	exp Lipids/ and (produc* or waste* or metaboliz*).mp.	243514
15	lipidom*.mp.	6393
16	((lipid or lipids) and (produc* or waste* or metaboliz*)).mp.	117010
17	(fatty acid* and (produc* or waste* or metaboliz*)).mp.	73084
18	(fatty acyl* and (produc* or waste* or metaboliz*)).mp.	1743
19	or/9-18	625384
20	8 and 19	1188
21	exp animals/ not (exp animals/ and exp humans/)	4780109
22	20 not 21	1097

Database(s): **Embase Classic+Embase** 1947 to 2021 January 25
Search Strategy:

#	Searches	Results
1	exp psoriasis/ [includes psoriatic arthritis]	98341
2	psorias*.mp.	92060
3	psoriat*.mp.	40006
4	(pustul* adj4 palm*).mp.	2611
5	(pustul* adj4 plantar*).mp.	435
6	(arthrit* adj4 mutilans).mp.	278
7	(pustul* adj4 soles).mp.	53
8	1 or 2 or 3 or 4 or 5 or 6 or 7	107911
9	exp lipidomics/ or exp metabolomics/	41255
10	metabolom*.mp.	52683
11	metabolit*.mp.	553048
12	(metabol* adj4 produc*).mp.	58482
13	(metabol* adj4 waste*).mp.	1494
14	exp lipid/ and (produc* or waste* or metaboliz*).mp.	346994
15	lipidom*.mp.	9526
16	((lipid or lipids) and (produc* or waste* or metaboliz*)).mp.	170118
17	(fatty acid* and (produc* or waste* or metaboliz*)).mp.	97017
18	(fatty acyl* and (produc* or waste* or metaboliz*)).mp.	1876
19	or/9-18	982610
20	8 and 19	2731
21	(exp animals/ or exp animal experimentation/ or nonhuman/) not ((exp animals/ or exp animal experimentation/ or nonhuman/) and exp human/)	7426943
22	20 not 21	2412

Database(s): **Cochrane Central Register of Controlled Trials** 2014 to Present
Search Strategy:

#	Searches	Results
1	exp psoriasis/ [includes psoriatic arthritis]	3351
2	psorias*.mp.	8368

3	psoriat*.mp.	3216
4	(pustul* adj4 palm*).mp.	167
5	(pustul* adj4 plantar*).mp.	25
6	(arthrit* adj4 mutilans).mp.	17
7	(pustul* adj4 soles).mp.	20
8	1 or 2 or 3 or 4 or 5 or 6 or 7	9589
9	exp lipidomics/ or exp metabolomics/	235
10	metabolom*.mp.	1879
11	metabolit*.mp.	14775
12	(metabol* adj4 produc*).mp.	1585
13	(metabol* adj4 waste*).mp.	26
14	exp Lipids/ and (produc* or waste* or metaboliz*).mp.	6176
15	lipidom*.mp.	256
16	((lipid or lipids) and (produc* or waste* or metaboliz*)).mp.	6773
17	(fatty acid* and (produc* or waste* or metaboliz*)).mp.	3489
18	(fatty acyl* and (produc* or waste* or metaboliz*)).mp.	2
19	or/9-18	27957
20	8 and 19	140

BIOSIS Citation Index
January 26, 2021

**Set**	**Results**	**Save History / Create AlertOpen Saved History**
# 3	**476**	#2 AND #1<br>*Indexes=BCI Timespan=All years*
# 2	**338,667**	TS=(metabolom* or metabolit* or lipidom* or ((metabol* or lipid* or fatty acid* or fatty acyl*) NEAR4 (produc* or waste* or produc* or metaboliz*)))<br>*Indexes=BCI Timespan=All years*
# 1	**44,339**	TS=(psorias* or psoriat* or (pustul* NEAR4 (palm* or plantar* or mutilans or soles)))<br>*Indexes=BCI Timespan=All years*
